# An Unusual Form of Choanal Atresia in a Full-term Newborn

**DOI:** 10.7759/cureus.11437

**Published:** 2020-11-11

**Authors:** Azhar A Sh. Hassan, Zainab S Bumuzah, Sara A Alomar, Ghadir A Alwabari, Zahra A AlAli, Zahra Y Al Abbas, Wasan M Alharbi, Shouq A Alraddadi, Dinah A AlNoaimi, Alaa K Alshammari

**Affiliations:** 1 Family and Community Medicine, Imam Abdulrahman Bin Faisal University, Dammam, SAU

**Keywords:** choanal atresia, infant respiratory distress, atrial septal defect secundum

## Abstract

Choanal atresia is a rare developmental condition that is defined as a narrowing or complete blockage of the nasal passages. Rapid surgical management is crucial in cases of bilateral choanal atresia since it may develop into a life-threatening emergency. We present the case of a full-term female newborn who developed mild respiratory distress soon after birth. The pediatrician was not able to insert a feeding tube through the nostrils despite repeated attempts. Cranial computed tomography confirmed the diagnosis of bilateral choanal atresia with an ectopic nostril. Furthermore, echocardiography demonstrated moderate atrial septal defect. The newborn underwent a successful correction of this anomaly via the trans-nasal surgical approach.

## Introduction

Choanal atresia is defined as a failure of the nasal cavity to communicate with the nasopharynx. This developmental anomaly was first described by Roederer in 1755 [1]. It is a rare congenital anomaly that is twice as common in female newborns as it is in male newborns [2]. This condition can be bilateral in 40% of the cases. It is thought that choanal atresia develops as a result of the persistence of the oronasal membrane which prevents the joining of the nasal and pharyngeal cavities. However, this theory does not explain the associated midface anomalies. An alternative theory suggests that choanal atresia arises due to dysregulations in the growth factors [3].

A rapid diagnosis and surgical management are crucial since this condition may develop into a life-threatening emergency. While choanal atresia may present as an isolated congenital anomaly, sometimes other anomalies such as cardiac malformations may co-occur with it [4]. Herein, we report the case of a full-term newborn with bilateral choanal atresia and ectopic nostril that was associated with a moderate atrial septal defect.

## Case presentation

We report the case of a female newborn, second child of a non-consanguineous couple, born at 41 weeks of gestation by spontaneous vaginal delivery, weighing 3400 g. The Apgar scores were seven and eight at one and five minutes, respectively. The pregnancy was without any complication and there was no history of exposure to teratogens. Both parents had no previous medical illnesses or relevant family history.

The baby was noted to have mild respiratory distress as evidenced by tachypnea and suprasternal retractions. The oxygen saturation was 92% on room air. Clinical examination revealed an abnormal opening below the right nostril. The pediatrician was not able to insert a feeding tube through the nostrils despite repeated attempts. A chest radiograph revealed normal findings.

**Figure 1:**
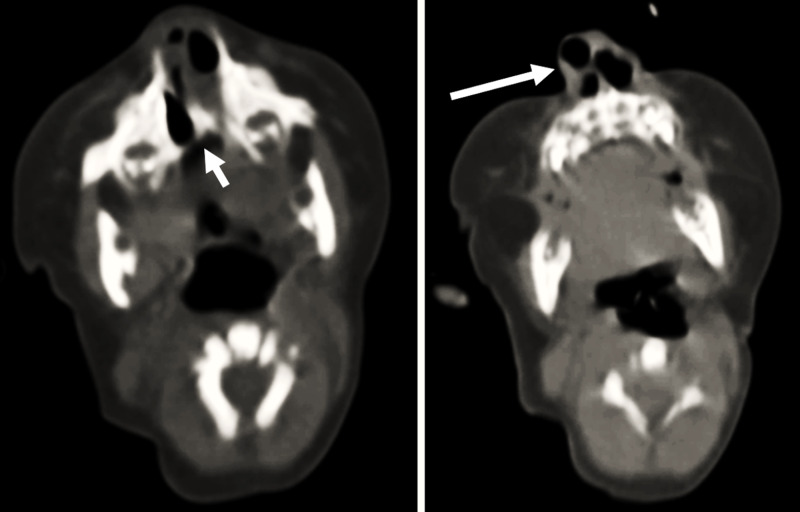
Cranial CT Cranial CT images demonstrating the bilateral choanal atresia (short arrow) and the right ectopic nostril (long arrow).

A cranial computed tomography scan confirmed the diagnosis of bilateral choanal atresia with ectopic right nostril (Figure 1). The Otorhinolaryngology Team was consulted and corrective surgery was planned after performing a survey of other systems for any associated congenital anomalies.

**Figure 2:**
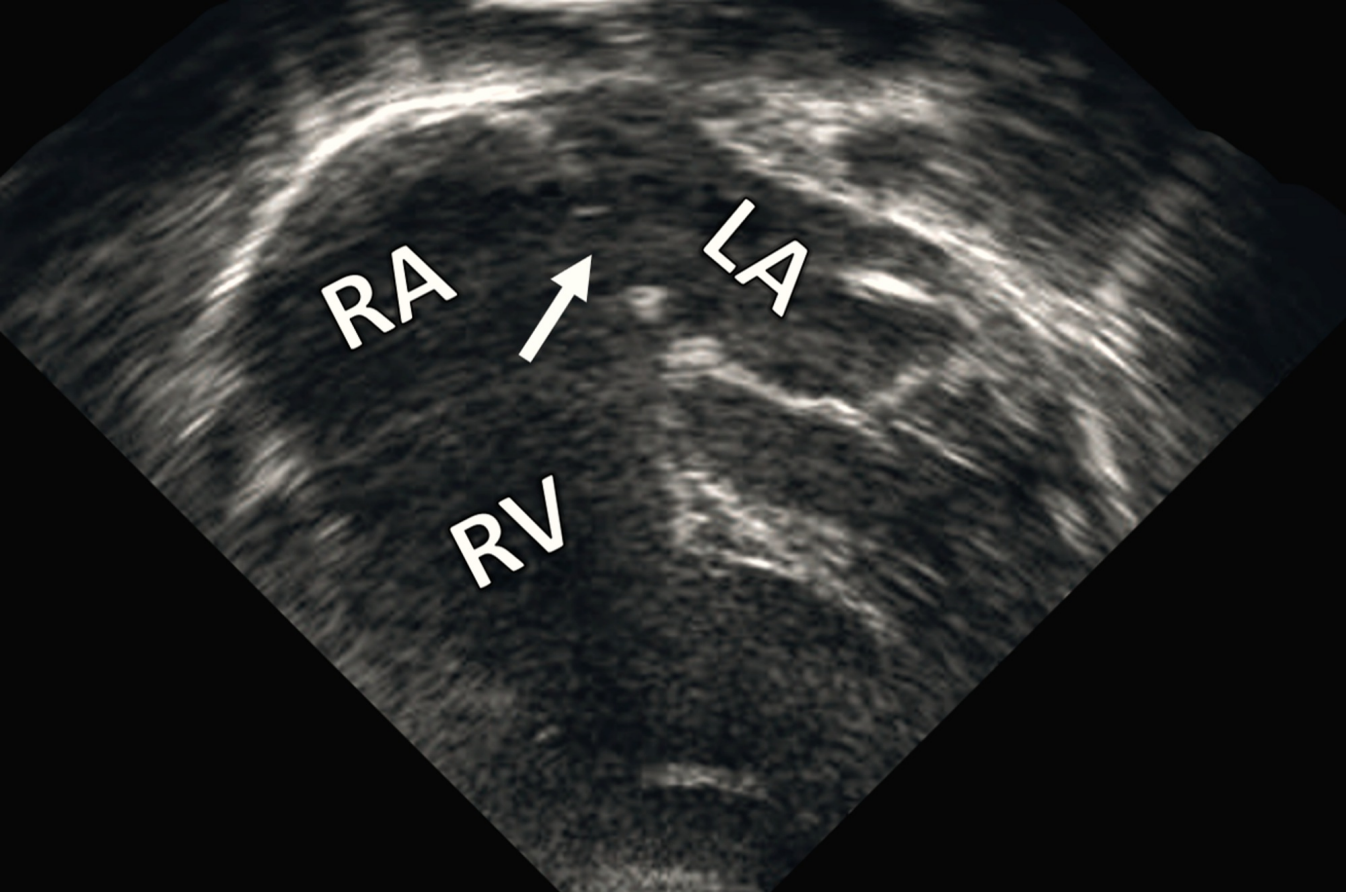
Echocardiography Echocardiographic image showing a moderate atrial septal defect (arrow). RA: right atrium; LA: left atrium; RV: right ventricle.

Bedside echocardiography revealed a moderate atrial septal defect with normal systemic and pulmonic circulations (Figure 2). Abdominal ultrasonography was normal.

**Figure 3:**
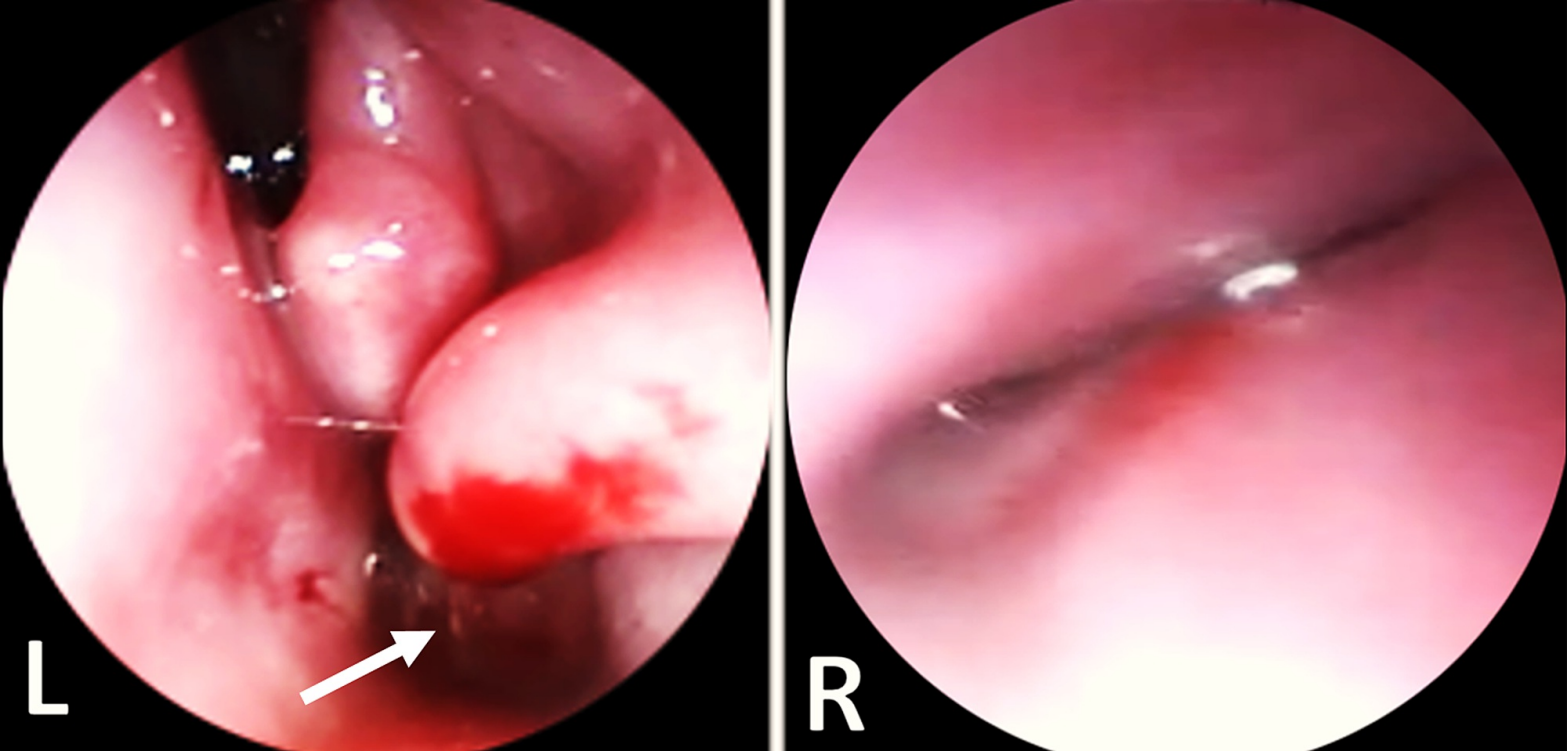
Operative findings Endoscopic images showing complete choanal atresia (arrow) on the left (L) and an ectopic, blind-ended nasal cavity with an absent nasal turbinate on the right (R).

On the fifth postnatal day, the baby underwent the operation. She was confirmed to have complete choanal atresia on the left and an ectopic, blind-ended nasal cavity with an absent nasal turbinate on the right (Figure 3). The hard and soft palates were normal.

Noradrenaline was infiltrated to the posterior segment of the septum through the left nasal cavity. Using the zero-degree 2.7-mm sinus telescope, a puncture was made in the left atretic posterior choana. Both the right and left posterior nasal choanae were widened using backbiting. The posterior part of the vomer bone was then removed to make both choanae to communicate in a common posterior choana. A microdebrider was used to trim the edges at the common choana. The ectopic, blind-ended, right nostril was left untouched.

No stent or nasal packing was placed. The operation course was without complication. The baby received a single dose of antibiotic prophylaxis. Suctioning and nasal drops were used regularly following the surgery. Follow up visits revealed no recurrent obstruction or stenosis of the nasal passages.

## Discussion

Choanal atresia is defined as a narrowing or complete blockage of the nasal passages. It is a developmental disorder related to the migration of the neural crest cells. Choanal atresia is a rare condition that is seen in around 8 per 100,000 live births. It has different types, including bony, membranous, or mixed type atresia [5].

Newborns are obligate nasal breathers. Bilateral choanal atresia is often present with signs of respiratory distress that are relieved during crying [1]. Hence, the diagnosis of bilateral choanal atresia should be considered in newborns with severe respiratory distress and absent nasal flaring. Choanal atresia is associated with a wide spectrum of congenital anomalies, including the CHARGE (coloboma, heart defect, atresia choanae, retarded growth, genitourinary abnormalities, and ear anomalies) association. It is also associated with chromosomal and gene defects. Such associations are found to be more common in bilateral choanal atresia compared with unilateral cases [6].

In the current case, the presence of an abnormal opening below the right nostril raised the suspicion of bilateral choanal atresia. Furthermore, the newborn did not develop signs of severe respiratory distress such as cyanosis due to the ectopic right nostril which had a narrow communication with the posterior choana. However, computed tomography remains the diagnostic modality of choice for choanal atresia [7].

Different approaches have been proposed for the management of choanal atresia [5]. We have used the trans-nasal route to avoid unfavorable blood loss and to preserve the growing palate by avoiding any damage to the craniofacial structures.

We did not place any stent after the surgery since we maintained a wide common posterior nasal choana by removing part of the vomer bone. The risk of stenosis after this maneuver is minimal. Furthermore, this maneuver helps in avoiding complications like a foreign-body reaction, skin necrosis, septal-cartilage necrosis, and perforation.

It is essential to note that choanal atresia might be associated with other congenital anomalies [4]. In the present case, a moderate atrial septal defect was the only associated condition.

## Conclusions

The presence of ectopic nostrils should raise the suspicion of bilateral choanal atresia in newborns with respiratory distress. A multidisciplinary approach is of paramount importance in the management of choanal atresia. A careful evaluation of other systems is essential because of the associated congenital anomalies.
